# A linear indurated forehead lesion with scalp alopecia

**DOI:** 10.1016/j.jdcr.2026.03.006

**Published:** 2026-03-13

**Authors:** Wan-Hao Tsai

**Affiliations:** a Division of Immunology and Rheumatology, Fu Jen Catholic University Hospital, New Taipei City, Taiwan; b Division of Rheumatology, Immunology, and Allergy, Department of Internal Medicine, National Taiwan University Hospital, Taipei, Taiwan

**Keywords:** cicatricial alopecia, en coup de sabre, forehead lesion, linear morphea, localized scleroderma

## Case description

A 43-year-old man presented with a gradually progressive, asymptomatic lesion on the forehead that had developed over approximately 1 year. He denied headache, seizures, visual disturbances, or other neurologic symptoms. There was no history of Raynaud phenomenon, sclerodactyly, digital ulcers, or internal organ involvement.

**Fig 1:**
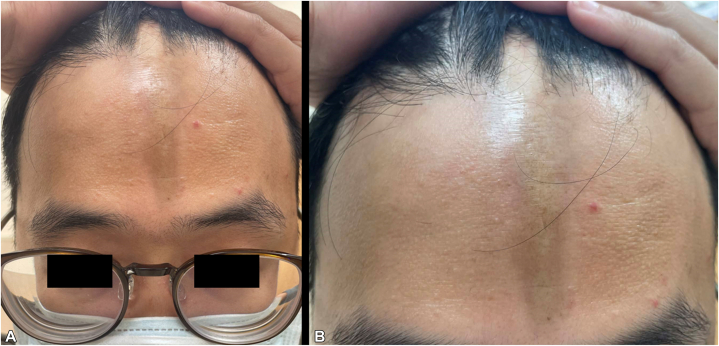
**A,** A sharply demarcated, linear, hyperpigmented, indurated depression with a subtle surrounding erythematous rim extending vertically along the paramedian forehead. **B,** Extension of the lesion into the frontal scalp, resulting in localized cicatricial alopecia.

Physical examination revealed a linear, hyperpigmented, indurated depression extending vertically along the paramedian forehead into the frontal scalp, with a subtle surrounding erythematous rim (Fig 1). The lesion followed a strikingly linear configuration and was associated with localized cicatricial alopecia. No facial asymmetry or hemifacial atrophy was observed.

Laboratory investigations were unremarkable, and there was no clinical or serologic evidence suggestive of systemic sclerosis. Nailfold capillaroscopy demonstrated preserved capillary density and architecture without dilatation, capillary dropout, or hemorrhage. Magnetic resonance imaging of the brain revealed no intracranial abnormalities.

Treatment with methotrexate in combination with a short course of systemic corticosteroids resulted in gradual softening of the lesion and partial improvement in skin induration.

**Question: What is the most likely diagnosis?**

**A.** Linear lichen planus
**B.** Parry–Romberg syndrome
**C.** En coup de sabre (linear morphea)
**D.** Discoid lupus erythematosus
**E.** Frontal fibrosing alopecia

Correct answer: **C.** En coup de sabre (linear morphea)

## Discussion

The correct diagnosis is en coup de sabre, a linear subtype of localized scleroderma (morphea) characterized by a band-like area of cutaneous sclerosis involving the frontoparietal region. The name derives from the resemblance of the lesion to a scar caused by a sword strike. Typical features include linear induration, pigmentary change, and possible involvement of underlying soft tissue or osseous structures, including the scalp, leading to cicatricial alopecia.^1^

Although en coup de sabre is more frequently described in pediatric populations, adult-onset cases are increasingly recognized. In contrast to systemic sclerosis, localized scleroderma is not associated with Raynaud phenomenon, sclerodactyly, nailfold capillary abnormalities, or internal organ involvement.^2^ Although autoantibodies may be present in localized scleroderma,^3^ the distinction from systemic sclerosis is made clinically rather than serologically.

Neurologic and ophthalmologic involvement has been reported in association with en coup de sabre, even in patients without overt neurologic symptoms.^4^ Accordingly, brain magnetic resonance imaging is recommended in patients with morphea affecting the head and neck region, regardless of neurologic symptoms.^1^ Baseline ophthalmologic evaluation should be considered in patients with linear morphea involving the head and neck because of potential ocular involvement.^5^ Neurologic and ophthalmologic complications are more frequently reported in pediatric-onset disease, although adult-onset cases may also develop extracutaneous involvement.^6^ Early recognition and treatment of active disease are important to limit disease progression and irreversible tissue damage. Systemic immunosuppressive therapy, including methotrexate with or without corticosteroids, remains first-line treatment for active disease, while mycophenolate mofetil may be considered in patients who fail or cannot tolerate methotrexate.

## Conflicts of interest

None disclosed.
